# Circulating MMP-12 as Potential Biomarker in Evaluating Disease Severity and Efficacy of Sublingual Immunotherapy in Allergic Rhinitis

**DOI:** 10.1155/2022/3378035

**Published:** 2022-06-12

**Authors:** Yandan Zhou, Min Xu, Wei Gong, Xiaoming Kang, Renzhong Guo, Jie Wen, Dinggang Zhou, Manjing Wang, Dazhi Shi, Qiancheng Jing

**Affiliations:** ^1^Changsha Aier Eye Hospital, Aier Eye Hospital Group, Changsha, Hunan 410000, China; ^2^Department of Otolaryngology Head and Neck Surgery, The Affiliated Changsha Central Hospital, Hengyang Medical School of University of South China, Changsha, Hunan 410000, China; ^3^Department of Otolaryngology Head and Neck Surgery, The Second Affiliated Hospital, Hengyang Medical School of University of South China, Hengyang, Hunan 421001, China

## Abstract

**Background:**

Allergic rhinitis (AR) is a highly heterogeneous disease, and allergen-specific immunotherapy (AIT) is an effective treatment. This study aims to evaluate the circulating mas-related G protein-coupled receptor-X2 (MRGPRX2) and matrix metalloproteinase-12 (MMP-12) levels in evaluating disease severity and predicting efficacy of SLIT in AR patients.

**Methods:**

We enrolled 110 moderate-severe persist AR patients (AR group) and 40 healthy controls (HC group). Circulating levels of MRGPRX2 and MMP-12 were measured, and their associations with disease severity were evaluated. All AR patients were assigned to receive sublingual immunotherapy (SLIT), and the efficacy was evaluated, and serum samples were collected at 1 year and 3 years after treatment. The correlations between serum MRGPRX2 and MMP-12 and clinical efficacy were assessed.

**Results:**

The serum concentrations of MRGPRX2 and MMP-12 were significantly higher in the AR group than the HC group, and the elevated MMP-12 levels were correlated with VAS and TNSS, and serum MRGPRX2 levels were correlated with VAS. Finally, 100 and 80 patients completed 1-year and 3-year follow-up and were classified into effective and ineffective groups. Serum MRGPRX2 and MMP-12 levels were lower in the effective group than the ineffective group. Although serum MRGPRX2 and MMP-12 levels did not significantly change after 1 year SLIT, serum MMP-12 levels were decreased 3 years post-SLIT than baseline and 1 year post-SLIT levels. Receiver operating characteristic (ROC) showed that serum MMP-12 was a potential biomarker for predicting the efficacy of SLIT.

**Conclusion:**

Serum MRGPRX2 and MMP-12 appeared to be promising biological indicators in reflecting disease severity in AR patients. Moreover, circulating MMP-12 might serve as a reliable predictor for clinical responsiveness of SLIT.

## 1. Introduction

Allergic rhinitis (AR) is a chronic inflammatory disease of the nasal mucosa caused by immunoglobulin E (IgE)-mediated hypersensitivity responses after exposure to inhaled allergens [1, 2]. Recently, the prevalence of AR continued to increase and brought heavy socioeconomic and healthy burdens [3, 4]. Prior publications demonstrated that AR was a heterogeneous clinical disease with a broad spectrum of severity, and limited indicators or biomarkers have been presented to monitor the disease progression and disease severity [5]. Evaluating disease severity utilizing biomarkers contributed to understand the underlying mechanisms of AR and monitor the therapeutic effect [6, 7]. Therefore, exploring an accurate and reliable biomarker to assess AR severity is essential. Currently, medications and allergen-specific immunotherapy (AIT) is the mainstream therapies for AR, and AIT is the only treatment which can regulate the natural progression [8]. AIT can be administered by subcutaneous immunotherapy (SCIT) or sublingual immunotherapy (SLIT), but most AR patients prefer to choose SLIT because of its efficacy, safety, and convenience [9]. However, not all AR patients benefit from SLIT, and its effectiveness rate always fluctuates between 50.0% and 80.0% with inconsistent definitions of efficacy [10]. Therefore, it is clinically significant to explore objective indicators or strategies to early predict the efficacy of SCIT before its onset.

Mas-related G protein-coupled receptor-X2 (MRGPRX2), a biomarker expressed in mast cells, is an endogenous receptor associated with IgE-independent activation of mast cells. It was demonstrated that MRGPRX2 exhibited the specificity to induce mast cell degranulation and modulate inflammatory response and was involved in allergic and inflammatory diseases [11, 12]. Besides, recent studies found that serum MRGPRX2 levels were significantly elevated in asthma patients, and it could serve as a biomarker for predicting treatment outcomes [13]. Matrix metalloproteinase-12 (MMP-12), a 54-kDa preproenzyme, can be induced by M2 macrophages, and it contributes to promote the proliferation of Th2 cells and the secretion of IL-4 and IL-13, which is involved in the immune response [14, 15]. Prior publications indicated that MMP-12 was a pivotal regulator in the pathophysiology of several chronic inflammatory diseases, including asthma [16], arthritis [17], and multiple sclerosis [18]. Lygeros et al. [19] presented that the mRNA and protein levels of MMP-12 were enhanced in the tissue samples of chronic rhinosinusitis with nasal polyp patients in comparison with healthy controls. These evidences demonstrated that MRGPRX2 and MMP-12 might affect T cell proliferation and differentiation and participate in the regulation of immune responses. Therefore, we hypothesize that MRGPRX2 and MMP-12 may play significant roles in the pathological mechanisms of AR and contribute to the therapeutic effect of AIT. In this study, we aim to evaluate the potentiality of serum MRGPRX2 and MMP-12 as novel biomarkers for evaluating disease severity and predicting the clinical efficacy of SLIT in AR patients.

## 2. Methods

### 2.1. Study Design and Participants

One hundred and ten moderate-severe house dust mite (HDM)-induced AR adult patients who visited our department between October 2018 and January 2019 were consecutively recruited in this study. Forty age and sex-matched healthy volunteers without any allergic diseases were enrolled as healthy controls (HC). All AR patients met the diagnostic criteria provided by the Allergic Rhinitis and its Impact on Asthma (ARIA) guidelines with positive results of skin tests and/or serum-specific IgE [20]. These AR patients were excluded for the following reasons: accompanied with other immune or inflammatory diseases or severe renal, hepatic, or cardiac dysfunction; age<18 years; pregnancy or potential pregnancy; and a history of immunotherapy or systemic steroid or antiallergy drug consumptions within 4 weeks before registration. HCs were excluded from this study if they were in immunotherapy and systemic steroid or antiallergy treatments and had severe heart and kidney dysfunction. Clinical and demographic data were collected from all participants, including age, gender, body mass index (BMI), visual analogue scale (VAS), and total nasal symptom score (TNSS). The present study was approved by the Medical Ethics Committee of the Affiliated Changsha Central Hospital, Hengyang Medical School of University of South China. All participants provided informed consent before their enrollments.

### 2.2. Serum Sample Collection and MRGPRX2 and MMP-12 Measurement

Blood samples were collected from each subject using 5-ml vacuum blood collection tubes. In AR patients who treated with SLIT, specimens were collected at baseline, 1 year and 3 years after SLIT, respectively. All harvested samples were coagulated at room temperature for 60 min to isolate the blood cells and then centrifuged at 3500 rpm for 20 min at 4°C, and the serum was collected and stored at -80°C for subsequent testing. Serum MRGPRX2 and MMP-12 levels were detected by enzyme-linked immunosorbent assay (ELISA) kit commercial (Multisciences, Hangzhou, China) according to the manufacturer's instructions, and the operator was blinded to the detailed data of patients.

### 2.3. Immunotherapy

All included AR patients voluntarily received HDM SLIT for 3 years to obtain the long-term efficacy as previously recommended [21]. The standardized Der f drops provided by the Wolwo Pharma Biotechnology Company (Zhejiang, China) were self-administered by patients following the manufacturer's recommended schedule. The first administration was conducted under the guidance and supervision of a physician. During the treatment schedule, compliance education and specialized guidance were performed to reduce the rate of drop-out and improve the rate of effective. All adverse reactions were recorded during the whole therapy period by physicians.

### 2.4. Clinical Efficacy Evaluation

All patients were scored for impairment in daily activities and sleep based on the severity and frequency of symptoms [22]. The medication score was calculated as previously described [6]. The combined symptom medication score (SMS) was defined as the sum of the symptom score and the medication score [23]. The efficacy evaluation was conducted as previously described, and “effective” was defined when patient obtained at least 30% reduction of SMS compared to baseline level; otherwise, “ineffective” was regarded.

### 2.5. Statistical Analysis

Normally distributed data were expressed as mean ± standard deviation (SD), and nonnormally distributed data were expressed as median and interquartile range. One-way analysis of variance (ANOVA) or Mann–Whitney *U* test was used for comparison among three groups, and the Student-Newman-Keuls (SNK) method was used for subsequent comparisons to determine the source of significance; Student *t*-test or Kruskal-Wallis *H* test was used for comparison between two groups. Categorical data were expressed as numbers (%) and compared using the chi-square test. Spearman's test was performed to investigate the correlation. Multivariate logistic regression analysis was used to identify independent predictors of clinical efficacy of SLIT. Receiver operating characteristic (ROC) curves were created, and the area under the curve (AUC), sensitivity, specificity, and critical values were assessed. In all tests, *P* < 0.05 was considered statistically significant. All statistical analyses and ROC analyses were performed by SPSS Statistics for Windows (version 25.0, IBM Corp., Armonk, NY, United States), and other graphs were constructed by GraphPad Prism (version 8.3.0, Software Inc. La Jolla, CA, United States).

## 3. Results

### 3.1. Basic Demographics and Characteristics of Participants

The demographic and clinical data of all participants are shown in Table 1. The VAS and TNSS scores were higher in the AR group than the HC group, but no statistical difference was found in sex, age, BMI, smoking, and drinking between HC group and AR group (*P* > 0.05).

### 3.2. Serum MRGPRX2 and MMP-12 Were Elevated in AR Patients and Associated with Disease Severity

**Figure 1:**
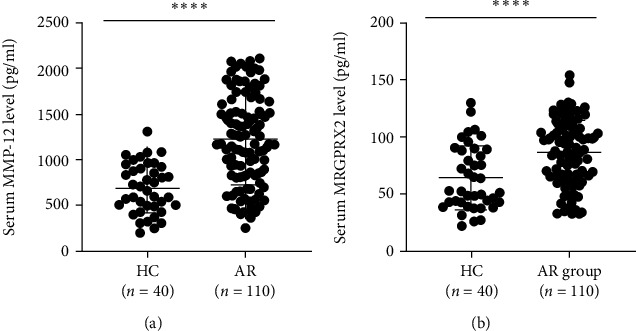
Serum MMP-12 and MRGPRX2 levels in the AR and HC groups. Serum MMP-12 (a) and MRGPRX2 (b) expression levels were significantly higher in the AR group than the HC group. MMP-12: matrix metalloproteinase-12; MRGPRX2: mas-related G protein-coupled receptor-X2; AR: allergic rhinitis; HC: health control. ∗∗∗∗*P* < 0.0001.

**Figure 2:**
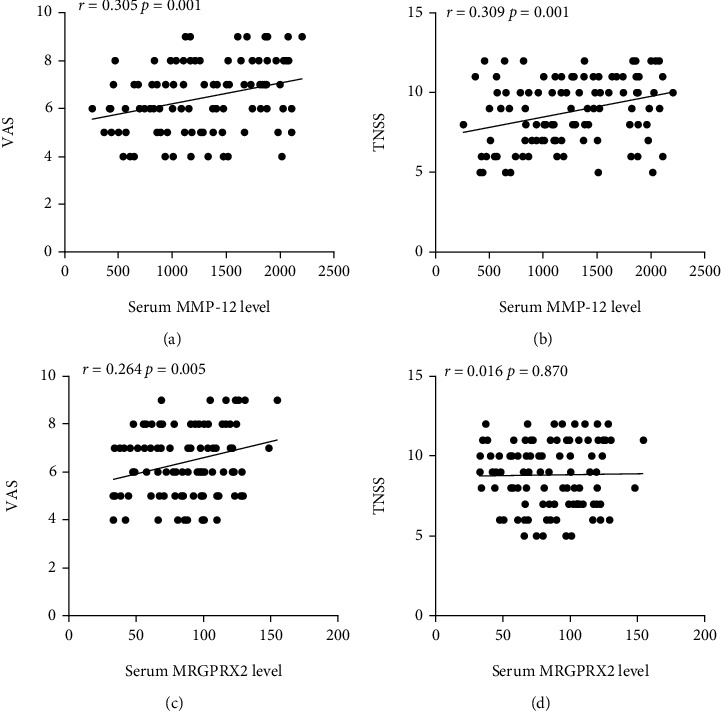
Associations between serum MMP-12 and MRGPRX2 levels and disease severity. Serum MMP-12 levels were positively correlated with VAS (a) and TNSS (b) scores, and MRGPRX2 levels were associated with VAS(c), but not with TNSS (d). MMP-12: matrix metalloproteinase-12; MRGPRX2: mas-related G protein-coupled receptor-X2; VAS: visual analogue scales; TNSS: total nasal symptoms score.

As presented in Figure 1, the serum MRGPRX2 and MMP-12 levels were markedly higher in the AR group than the HC group. Spearman's correlation analysis results showed that the serum MMP-12 levels were positively correlated with TNSS and VAS, and serum MRGPRX2 levels were positively associated with VAS (Figure 2). Detailed parameters are displayed in Table 2.

### 3.3. Serum MMP-12 Levels Associated with Clinical Efficacy of SLIT

**Figure 3:**
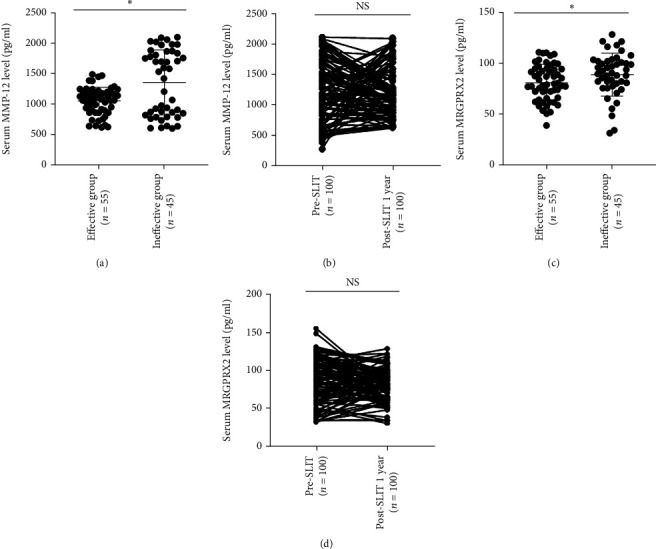
Serum MMP-12 and MRGPRX2 levels in two groups 1 year post-SLIT. (a) Serum MMP-12 levels were significantly lower in the effective group than the ineffective group, and concentrations were reduced after 1 year post-SLIT (b). (c) Serum MRGPRX2 level was significantly reduced in the effective group than the ineffective group, but the concentrations were not significantly reduced 1 year post-SLIT (d). MMP-12: matrix metalloproteinase-12; MRGPRX2: mas-related G protein-coupled receptor-X2; SLIT: sublingual immunotherapy, ∗*P* < 0.05; NS: no significance.

**Figure 4:**
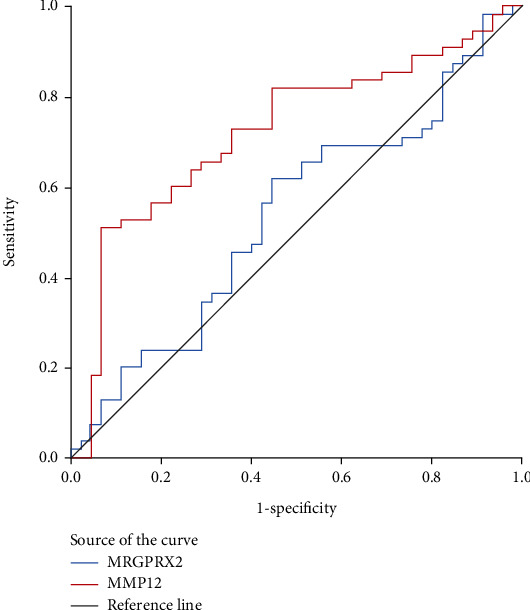
ROC curve analysis of MMP-12 and MRGPRX2 as promising biomarkers for predicting the efficacy 1 year post-SLIT in AR patients. ROC: receiver operating characteristic; MMP-12: matrix metalloproteinase-12; MRGPRX2: mas-related G protein-coupled receptor-X2; SLIT: sublingual immunotherapy; AR: allergic rhinitis.

**Figure 5:**
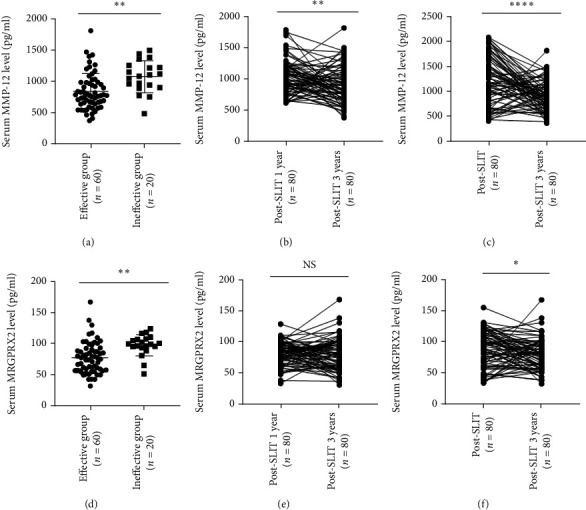
Serum MMP-12 and MRGPRX2 levels in the effective and ineffective groups 3 years post-SLIT. (a–c) Serum MMP-12 levels were significantly lower in the effective group compared to the ineffective group, and concentrations were significantly decreased after 3 years of SLIT than pre-SLIT and post-SLIT 1 year. (d–f) Serum MRGPRX2 levels were lower in the effective group than the ineffective group, and the concentrations were also decreased 3 years post-SLIT, but no statistical difference was observed between 3 years and 1 year post-SLIT. MMP-12: matrix metalloproteinase-12; MRGPRX2: mas-related G protein-coupled receptor-X2; SLIT: sublingual immunotherapy, ∗∗*P* < 0.01, ∗∗∗∗*P* < 0.0001, NS: no significance.

**Figure 6:**
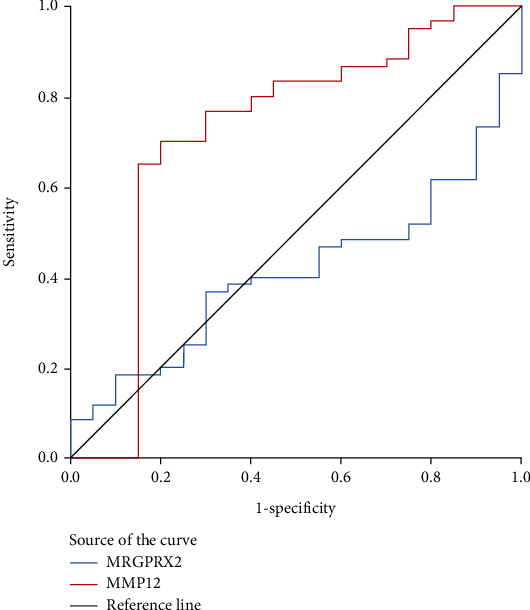
ROC curve analysis of MMP-12 and MRGPRX2 as promising biomarkers for predicting the efficacy 3 years post-SLIT in AR patients. ROC: receiver operating characteristic; MMP-12: matrix metalloproteinase-12; MRGPRX2: mas-related G protein-coupled receptor-X2; SLIT: sublingual immunotherapy; AR: allergic rhinitis.

After 1-year follow-up, 100 patients supplied complete data, and 55 patients were classified into effective group, and 45 patients were included into ineffective group, and demographic and clinical data are shown in Table 3. Serum MRGPRX2 and MMP-12 levels were lower in the effective group than the ineffective group, but their level were not statistically changed between baseline and 1 year post-SLIT (Figure 3). ROC curves in Figure 4 show that serum MMP-12 exhibited potential ability in predicting 1-year efficacy of SLIT, and detailed data are shown in Table S1. After 3-year follow-up, 80 patients provided complete data, and 60 patients were categorized into effective group, and 20 patients were included into ineffective group, and demographic and clinical data are presented in Table 4. As presented in Figure 5, serum MRGPRX2 and MMP-12 levels were decreased in the effective group than the ineffective group, and MMP-12 levels were significantly reduced 3 years post-SLIT than 1 year post-SLIT and baseline levels. Although serum MRGPRX2 levels were also decreased 3 years post-SLIT, no statistical difference was observed between 3 years and 1 year post-SLIT. ROC curves in Figure 6 demonstrate that serum MMP-12 was a promising biomarker in predicting 3-year efficacy of SLIT, but MRGPRX2 showed poor predictive ability. The detailed data are displayed in Table S2.

## 4. Discussion

In the current study, the results showed that the serum levels of MRGPRX2 and MMP-12 were increased in the AR group and positively correlated with TNSS and VAS scores, respectively. Furthermore, we demonstrated that serum MRGPRX2 and MMP-12 levels were associated with the efficacy of SLIT, and MRGPRX2 and MMP-12 levels were significantly decreased in the effective group than the ineffective group, and SLIT could significantly decrease their concentrations after 3-year treatment. ROC curves presented that MMP-12 exhibited good accuracy for predicting clinical efficacy of SLIT in AR patients. Taken together, these observations suggested that the serum MRGPRX2 and MMP-12 might be useful for evaluating the disease severity, and MMP-12 might serve as a reliable biomarker for predicting clinical efficacy of SLIT in AR patients.

Mast cells are multifunctional immune cells and well known for their roles in allergic and inflammatory diseases, including anaphylaxis, atopic dermatitis, and asthma [11, 23]. In addition, it was proven that mast cell degranulation was the crucial part in the pathological process of AR [24, 25]. MRGPRX2 is expressed predominantly in mast cells and activated by a broad range of cationic ligands, including substance P and hemokinin-1, which is subsequently involved in the development of allergic diseases [26, 27]. Moreover, a previous study demonstrated that MRGPRX2-mediated activation of mast cells may contribute to the pathogenesis of asthma [28]. In this study, our results showed that the levels of serum MRGPRX2 levels were enhanced in AR patients and positively correlated with VAS and TNSS scores. Thus, we speculated that enhanced mast cell degranulation and histamine release by high levels of MRGPRX2 contributed to aggravated allergic symptoms in AR patients. Similarly, MMP12, a proinflammatory factor mainly produced by macrophages, is demonstrated to be involved in the development of allergic diseases [14, 29]. Prior publications found that abnormal MMP expressions were implicated in various chronic inflammatory disorders, including asthma [30], chronic rhinosinusitis with nasal polyp [31], and inflammatory bowel disease [32]. Recent studies found that MMP12 was mainly secreted by M2-type macrophages, and the enhanced MMP-12 levels were proved to promote Th2-type inflammatory response and aggravated allergic airway inflammation in airway inflammatory diseases [14, 33]. Interestingly, the present study found that MMP12 levels were increased in AR patients and positively correlated with VAS and TNSS scores, which was in accordance with the previous conclusions. Given that, we have reasons to believe that MRGPRX2 and MMP12 are involved in the development of AR and associated with disease severity, but the potential mechanisms deserve further investigations.

Growing evidences proved that SLIT was an effective and safe treatment option and has been widely applied in moderate-severe AR patients [34, 35]. Although SLIT significantly alleviated allergic symptoms in AR patients, long-term follow-up results revealed that a quite proportion of patients did not benefit from this therapy [10, 36]. Therefore, exploring objective biomarkers to predict the efficacy of SCIT is particularly important. In the present study, we firstly found that serum MMP-12 levels were lower in the effective group than the ineffective group, and MMP-12 levels were gradually decreased with the increase of treatment time. Moreover, ROC curve analysis showed that serum MMP-12 was a credible and accurate biomarker for the predicting of SLIT efficacy both 1 year post-SLIT and 3 years post-SLIT. It is well known that dendritic cells as primary antigen-presenting cells act key roles in AR via promoting Th2 differentiation and regulating Th1/Th2 inflammation balance, and dendritic cells tolerance is considered as a pivotal indictor for reflecting the success of AIT [37–39]. MMP12 is expressed in dendritic cells and plays an essential role in the activation of and immune response [40, 41]. During the SLIT schedule, repeated stimulation of specific antigens may affect the function of dendritic cells and block the initiation of the immune response and then reduces the number of circulating allergen-specific Th2 cells [23, 38]. Combined with our findings, we can assume that the MMP-12 maybe a potential biomarker reflecting the activity of dendritic cells and contributes to the therapeutic effect of SLIT in AR patients, suggesting that serum MMP-12 can be used as an objective indicator to predict the clinical efficacy of SLIT. However, its clinical value and underlying mechanisms in AR patients need further explorations.

The current study has several limitations. First, the sample size is relatively small, and a validation cohort study is absent to strengthen the findings. Second, all participants were HDM-induced, and included in single medical center, which may limit the generalization. Further multicentric studies with larger sample sizes are needed to support and extend our current findings.

## 5. Conclusion

We indicated that the serum MMP-12 and MRGPRX2 levels were elevated in the AR patients and positively correlated with disease severity. We also found that serum MMP-12 exhibited potential ability to discriminate patients who respond to SLIT. These results suggest that serum MMP-12 was a useful and objective biomarker for evaluating the severity of AR and predicting the clinical efficacy of SLIT.

## Figures and Tables

**Table 1:** Demographics and clinical characteristics of patients between two groups.

Variables	AR group (n = 110)	HC group (n = 40)	*P* value
Sex			0.362
Male	54 (49.1%)	23 (57.5%)	
Female	56 (50.9%)	17 (42.5%)	
Age, years	28.3 ± 4.8	29.2 ± 4.0	0.287
BMI, kg/m^2^	23.7 ± 3.6	23.4 ± 3.6	0.626
Smoking, yes/no	53/57	15/25	0.245
Drinking, yes/no	49/61	23/17	0.160
Baseline VAS	6.4 ± 1.4	0	*<0.001*
Baseline TNSS	8.8 ± 2.1	0	*<0.001*

AR: allergic rhinitis; HC: health control; BMI: body mass index; VAS: visual analogue scales; TNSS: total nasal symptoms score.

**Table 2:** Association between and clinical variables in AR patients.

Variable	Serum MMP-12 level		Serum MRGPRX2 level	
	*r*	*P* value	*r*	*P* value
Age	-0.088	0.924	0.012	0.884
BMI	-0.071	0.386	0.065	0.428
Baseline VAS	0.305	*0.001*	0.264	*0.005*
Baseline TNSS	0.309	*0.001*	0.016	0.870
Serum MRGPRX2 level	0.239	*0.003*	—	—
Serum MMP-12 level	—	—	0.239	*0.003*

AR: allergic rhinitis; BMI: body mass index; VAS: visual analogue scales; TNSS: total nasal symptoms score; MMP-12: matrix metalloproteinase-12; MRGPRX2: mas-related G protein-coupled receptor-X2.

**Table 3:** Demographics and clinical characteristics of effective and ineffective group 1 year post-SLIT.

Variables	Effective group (n = 55)	Ineffective group (n = 45)	*P* value
Sex			0.077
Male	22 (40.0%)	26 (57.8%)	
Female	33 (60.0%)	19 (42.2%)	
Age, years	29.0 ± 4.8	27.4 ± 4.6	0.627
BMI, kg/m^2^	23.8 ± 3.6	23.2 ± 3.4	0.771
Smoking, yes/no	26/29	24/21	0.546
Drinking, yes/no	26/29	19/26	0.614
Baseline VAS	6.7 ± 1.4	6.1 ± 1.4	*0.027*
Baseline TNSS	9.3 ± 1.9	8.4 ± 2.2	0.202

BMI: body mass index; VAS: visual analogue scales; TNSS: total nasal symptoms score; SLIT: sublingual immunotherapy.

**Table 4:** Demographics and clinical characteristics of effective and ineffective group 3 years post-SLIT.

Variables		Effective group (n = 60)	Ineffective group (n = 20)	*P* value
Sex				0.515
Male		25 (41.7%)	10 (50.0%)	
Female		35 (58.3%)	10 (50.0%)	
Age, years		29.1 ± 4.9	26.8 ± 4.6	0.067
BMI, kg/m^2^		23.7 ± 3.5	22.6 ± 3.6	0.223
Smoking, yes/no		29/31	10/10	0.897
Drinking, yes/no		29/31	12/8	0.366
Baseline VAS		6.6 ± 1.4	5.8 ± 1.5	*0.034*
Baseline TNSS	9.3 ± 1.9	8.7 ± 2.0	0.766	

BMI: body mass index; VAS: visual analogue scales; TNSS: total nasal symptoms score; SLIT: sublingual immunotherapy.

## Data Availability

The data used to support the observations of this study are available from the corresponding author upon request.

## Supplementary Materials

Supplementary Materials Supplementary Materials Table S1: presented the detailed data of ROC curves of serum MMP-12 and MRGPRX2 in predicting 1year efficacy of SLIT. Table S2: displayed the detailed parameters of ROC curves of serum MMP-12 and MRGPRX2 in predicting 3 years efficacy of SLIT.
