# Supplementary material for: Circulating MMP-12 as Potential Biomarker in Evaluating Disease Severity and Efficacy of Sublingual Immunotherapy in Allergic Rhinitis
Source: Mediators Inflamm. 2022 Jun 12;2022:3378035. doi: 10.1155/2022/3378035 (PMC9207019; doi:10.1155/2022/3378035)
Supplement: Supplementary Materials — Table S1: presented the detailed data of ROC curves of serum MMP-12 and MRGPRX2 in predicting 1year efficacy of SLIT. Table S2: displayed the detailed parameters of ROC curves of serum MMP-12 and MRGPRX2 in predicting 3 years efficacy of SLIT. [file 3378035.f1.docx]

Table S1. ROC curves of serum MMP-12 and MRGPRX2 in predicting 1 year efficacy

| Variables | AUC (95% CI) | P value | cut-off value | sensitivity | specificity |
| --- | --- | --- | --- | --- | --- |
| Serum MMP-12 level | 0.723 (0.424-0.654) | **<0.001** | 1515.02 | 50.9% | 93.3% |
| Serum MRGPRX2 level | 0.539 (0.621-0.825) | 0.504 | 88.01 | 61.8% | 55.6% |

ROC: Receiver operating characteristic, AUC: the area under the curve, MMP-12: Matrix metalloproteinase-12, MRGPRX2: mas‐related G protein‐coupled receptor‐X2

TableS2. ROC curves of serum MMP-12 and MRGPRX2 in predicting 3 years efficacy

| Variables | AUC (95% CI) | P value | cut-off value | sensitivity | specificity |
| --- | --- | --- | --- | --- | --- |
| Serum MMP-12 level | 0.721 (0.570-0.872) | **0.003** | 939.64 | 70.0% | 80.0% |
| Serum MRGPRX2 level | 0.411(0.284-0.538) | 0.234 | 100.04 | 18.3% | 90.0% |

ROC: Receiver operating characteristic, AUC: the area under the curve, MMP-12: Matrix metalloproteinase-12, MRGPRX2: mas‐related G protein‐coupled receptor‐X2
